# Involving men in pregnancy: a cross-sectional analysis of the role of self-efficacy, gender-equitable attitudes, relationship dynamics and knowledge among men in Kinshasa

**DOI:** 10.1186/s12884-024-06638-1

**Published:** 2024-06-26

**Authors:** Francine E. Wood, Anastasia J. Gage, Eric Mafuta, Jane T. Bertrand

**Affiliations:** 1 Center On Gender Equity On Health, University of California San Diego, 9500 Gilman Drive, La Jolla, CA 92093 USA; 2 Department of International Health and Sustainable Development, School of Public Health and Tropical Medicine, Tulane University, 1440 Canal Street, New Orleans, LA 70112 USA; 3 School of Public, Health University of Kinshasa, Kinshasa, DR Congo

**Keywords:** Male involvement, Maternal health, Pregnancy, Decision-making, Gender-equitable attitudes, Self-efficacy, Relationship factors

## Abstract

**Background:**

Although male participation in maternal health has gained increasing recognition and support over the years, little is known about male involvement during pregnancy in the Democratic Republic of the Congo. This paper identified male involvement patterns during pregnancy and evaluated their associations with pregnancy and birth preparedness knowledge, gender-equitable attitudes, self-efficacy, and co-parental relationship factors. Lastly, it explored the moderating effect of gender-equitable attitudes and intimate partner violence on the association between relationship satisfaction and male involvement.

**Methods:**

Data from the 2018 Momentum baseline study were analyzed to determine the predictors of involvement. Factor analysis was used to create male involvement indices for antenatal carebirth preparedness and shared decision making. The sample consisted of 1,674 male partners of nulliparous pregnant women who were 6 months pregnant at baseline.

**Results:**

Male involvement in individual pregnancy-related activities was low, ranging from 11% (finding a blood donor) to 49% (saving money during emergencies). Knowledge of the number of antenatal care visits, birth preparedness steps, and newborn danger signs were positively associated with involvement in antenatal care/birth preparedness activities while knowledge of antenatal care benefits was positively associated with involvement in shared decisions. Increasing relationship satisfaction and self-efficacy were associated with antenatal care/birth preparedness involvement and for shared decisions, a positive association with gender-equitable attitude and a negative association with self-efficacy were observed. Moderation effects were also detected.

**Conclusions:**

The findings suggest that male involvement is multifaceted and factors influencing involvement vary depending on the type of involvement. Addressing these factors can improve male participation in maternal health.

**Supplementary Information:**

The online version contains supplementary material available at 10.1186/s12884-024-06638-1.

## Background

In many sub-Saharan African countries, men are usually key decision-makers, controlling and deciding on resources in the household, such as financial support [1]. This has implications for maternal health and evidence suggests that involving men in pregnancy, delivery and the post-delivery period can help reduce maternal and neonatal mortality [2]. Other positive benefits include increasing access to and use of maternal services and contraceptives, discouraging unhealthy maternal health practices and encouraging more equitable couple communication and decision making [2–5]. Male involvement in health care also benefits men themselves: they are healthier, more connected socially and have improved relationships with their partners [6]. In countries such as the Democratic Republic of the Congo (DRC), increasing involvement of male partners could potentially reduce maternal mortality among other potential benefits.

Involving men in maternal, neonatal and child health has received increased recognition over the years [7, 8]. Despite the increased focus on male involvement, there is no accepted standard definition of the concept. Studies have defined male involvement in various ways depending on the stage of pregnancy, the relationship with the pregnant woman and the study context. The most common measure has focused only on attendance at facility-based maternal health services [3, 9, 10], but it is acknowledged that the use of a single indicator, such as antenatal care (ANC) attendance, to measure male involvement is inadequate.

Some studies have utilized scales and indices to gauge male involvement at different stages of pregnancy [11–13]. In Kenya, for example, Mangeni et al. [14] used two measures to define male involvement: attendance at antenatal care visits and positive male perception of women's health. In Tanzania, a composite score was used to measure male involvement [11]. Men were given a score ranging from one to five, with five being the highest involvement, based on whether or not they (a) escorted their wives to antenatal care, (b) escorted their wives to delivery, (c) had shared decision-making on where to deliver, (d) knew at least three danger signs of pregnancy, childbirth and postpartum, and (e) had taken at least three birth preparedness and complication readiness actions [11]. More recently, in Kenya, a two-factor structure (male encouragement/reminders and active participation) scale was used to measure male involvement [13].

Notwithstanding the lack of uniformity in the measurement of involvement, the prevalence of male involvement is low in the DRC as well as in other sub-Saharan African countries. A review of the literature found two studies in the DRC that measured the prevalence of male involvement in pregnancy-related activities [15, 16]. In a randomized control trial by Ditekemena et al. [16], one in five men attended HIV counseling and testing during the pregnancy period. An even lower prevalence (7%) was measured in the Malamu project, where male partners were invited to clinics using invitation letters given to women attending antenatal care (ANC) services. Male partners who attended ANC with their partners were also tested for HIV; testing of male partners increased from four percent to seven percent over the course of the project [15]. Studies in other sub-Saharan African countries also found low rates of male involvement ranging from 11% to 60% in various pregnancy-related activities [17, 18].

The low to moderate levels of involvement are shaped by many factors, including education, relationship status, social and gender norms, and the lack of attention to men in maternal, neonatal and child health policies [19, 20]. Each factor influences the involvement of the male partner differently. For instance, social expectations of gender roles influence men’s participation in pregnancy-related activities [19, 21, 22], whereby men who did not perceive antenatal care as a woman’s domain were more likely to be involved. Self-efficacy and a man’s attitude to gender norms also influence involvement in various maternal and child health activities [19, 23]. The DRC is a populous and highly diverse country with many ethnic groups and kinships, and the diversity can lead to various perceptions of gender norms and roles [24]. Recent work suggests that matrilineal kinship undermines spousal cooperation, where matrilineal individuals tend to cooperate less with their spouses [25], resulting in greater inefficiencies in the household. Studies have also found that good couple communication was associated with male partner support [26, 27], and weaker relationships deterred involvement [22]. Ultimately, kinship could influence the opportunity and desire of the male partner to be involved in pregnancy.

Given the importance of male involvement and the dearth of literature on male involvement in pregnancy in the DRC, there is a need for further research on this topic. In addition, most relevant studies in the DRC and sub-Saharan Africa have been conducted as part of HIV/AIDS interventions [10, 28]. An improved understanding of the determinants of male involvement in pregnancy-related activities outside the realm of HIV programming could potentially inform intervention strategies. Furthermore, it can aid in developing programs and policies that encourage male participation in maternal health and guide future research. Although the recent body of research has used more comprehensive measures of male involvement, prior studies conducted in the DRC explored the behavior as a binary measure focused primarily on attendance at antenatal care visits. The binary nature does not capture the multidimensionality of the behavior and does not focus on involvement outside of the health facility.

To this end, the present study used multiple indicators to define male involvement during the first-six months of pregnancy in the DRC and examined the determinants associated with male involvement during pregnancy. Another objective was to explore the influence of deterrents of involvement on male partners with strong relationships with first-time mothers (FTMs). Some studies suggest that perceptions of pregnancy-related activities as a “woman’s domain” and intimate partner violence are barriers to involvement [19, 22], while others find that good couple communication and strong spousal/partner relationships facilitated involvement [22, 26, 27]. Therefore, male partners who are satisfied with their relationship and do not commit acts of violence or have favorable gender-equitable attitudes could be more involved. For this last objective, we hypothesized that the association between relationship satisfaction and involvement would be augmented by positive gender-equitable attitudes and reduced by violence perpetration.

### Conceptual framework

**Fig. 1:**
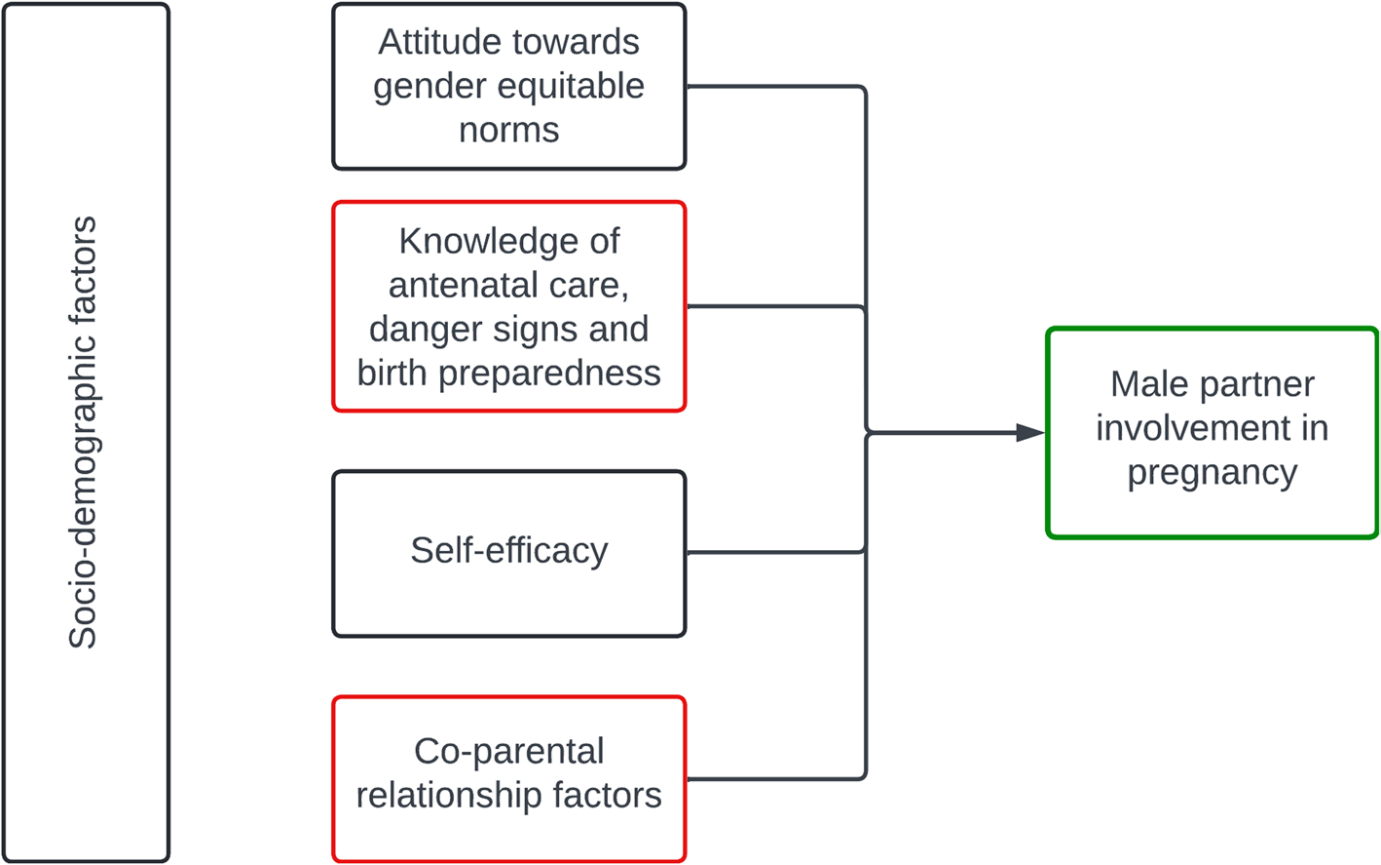
Conceptual model of the predictors of male partner involvement in pregnancy-related activities during the first six-months of pregnancy. Note: The red boxes show the primary factors of interest informed by the responsible fatherhood framework. Some of the factors informed by the responsible fatherhood framework are included in the socio-demographic characteristics

The conceptual framework illustrated in Fig. 1 guided the analysis of the association of attitude towards gender-equitable norms, knowledge, co-parental relationship factors, and self-efficacy with male involvement in pregnancy. It drew upon father and co-parental relationship factors identified in the Responsible Fatherhood Framework [29] and was expanded to include factors – such as attitudes towards gender norms and self-efficacy – identified in the literature and available in the dataset.

## Methods

### A. Data and population

The analysis was based on cross-sectional data from the Momentum Project baseline survey conducted by Tulane University School of Public Health and Tropical Medicine from September to November 2018. Momentum was a three-year gender-transformative integrated family planning, maternal and newborn health, and nutrition intervention in Kinshasa, the capital city of the DRC. The intervention used home visits, community dialogue, and support group education sessions to improve care-seeking and maternal and neonatal health (MNH) and nutrition practices, increase the use of postpartum family planning methods, and increase gender-equitable attitudes and behaviors. The project’s survey sample was drawn from a purposive sample of FTMs aged 15 to 24 who were approximately six-months pregnant at the time of the baseline survey and their male partners residing within intervention (Kingasani, Lemba, and Matete) and comparison (Bumbu, Ndjili, and Masina 1) health zones in Kinshasa. Additional details on Momentum and findings on the project’s impact among the FTMs and male partners have been published elsewhere [30–35].

To be enrolled in the study, FTMs and their male partners had to be: (1) willing and mentally competent to provide consent; (b) able to speak Lingala or French; and (3) reside permanently (i.e., not visiting) in the study health zones. In addition to the above, FTMs had to be approximately six-months pregnant with their first child at baseline and male partners had to be the husband or partner of a recruited FTM.

Trained interviewers used a pre-tested questionnaire to ask eligible male partners about their background characteristics; contraceptive knowledge, norms, attitudes, and practices; fertility desires; attitudes and behaviors in various aspects of pregnancy and newborn health; gender-equitable attitudes and behaviors; relationship satisfaction with the FTM; perceived social norms regarding male participation in childcare; and intimate partner violence perpetration. The data were collected via smartphones and many of the questions on the survey were informed by the findings from the formative research.

Out 2,088 male partners identified, 1,769 were eligible, provided written consent and were completely interviewed in the baseline survey. The final sample used in this analysis consisted of male partners who were interviewed at baseline and the analysis was restricted to those with no missing data on any of the variables included in the analysis. Of the 1,769 male partners interviewed, 92 did not have data on specific characteristics of FTMs used for the analysis and of the 1,674 male partners with complete data, 213 did not have adequate privacy and were not asked questions about intimate partner violence (IPV) perpetration.

#### Comparison of participants with missing data

As shown in Table S6 in Additional File 1, male partners with missing data were slightly older (28 years) than those without missing data (27 years), but the difference was not statistically significant. For the remaining background characteristics, significant differentials were observed for three characteristics. Significantly more male partners with missing data were ever married, lived in Bumbu and Masina 1, and had lived in the health zone of residence for less than five years.

#### Ethical approval

Ethical approval was obtained from the Tulane University Institutional Review Board (2018–1028) and the University of Kinshasa School of Public Health Ethics Committee (ESP/CE/066/2018). Additionally, written informed consent was obtained from all survey participants on paper and via smartphone before the start of data collection.

### B. Measures

#### Outcome variable

The outcome variable, male involvement during pregnancy – specifically during the first six-months of pregnancy – was measured by collecting information on the male partner’s participation in ANC-related activities (Table 1). Male partner involvement was explored as two continuous variables, focusing on the number of ANC and birth preparedness activities and shared decisions in which men participate. From a programmatic standpoint, analyzing involvement by exploring the effect of each additional pregnancy-related activity may be more informative and meaningful than analyzing involvement as levels (low, level and high).

**Table 1:** Description of items measured in the male involvement composite score

	**Survey Question**	**Response option**	**Definition of involvement**^**a**^
Participation in antenatal care-related activities	Please tell me if you [male partner] have participated in the following things for [NAME OF FTM’s] pregnancy: 1. finding information about the pregnancy 2. making decisions about antenatal care 3. making a birth plan 4. saving money for emergencies 5. arranging transport for delivery 6. deciding on skilled attendance at delivery 7. arranging for a blood donor	No/Yes	Present
	Were you present during any of those antenatal check-ups?	Present/ Not present	Yes
Participation in decision making	Would you say that the following are mainly your decision, mainly [name of first-time mother (FTM)]’s decision, someone else’s decision or did you and [name of FTM] decide together? 1. where to deliver the baby 2. when to seek care and treatment for danger signs of the mother and newborn 3. where to seek care and treatment for danger signs of the mother and newborn	Respondent and first-time mother jointly, someone else	Respondent and first-time mother jointly

^a^NB: all other response categories not indicated were categorized as no involvement

Prior to the formation of the composite score, exploratory factor analysis (EFA) was used to examine and identify the structure and dimensions of the score. The Kaiser–Meyer–Olkin (KMO) test and the Bartlett Test of Sphericity were calculated before conducting the EFA to ensure the appropriateness of EFA. The internal consistency of the items was assessed using Cronbach’s alpha. The KMO results (0.858) indicated that the variation in the data was well suited to EFA, and the correlations among the variables were significant (Bartlett test *p*-value = 0.000). The factor analysis with rotation yielded two unique factors with eigenvalues of 3.54 and 1.41 and explained 79.5% and 30.4% of the variance, respectively. On both factors, all items, except one, had factor loadings greater than 0.3. Confirmatory factor analysis (CFA) was used to examine the construct validity of the male involvement scale, and the results support a two-factor model as suggested in the EFA (Table S1 in Additional File 1) [36, 37].

Based on the factor analysis findings, two domains were identified and constructed by summing all the items identified in each factor. The first, involvement in ANC and birth preparedness, ranged from 0 – 7 (α = 0.8602), and the second, participation in shared decision making ranged from 0 – 3 (α = 0.7272).

For a subset of our analysis, the scores were further divided into categories – low, medium, and high – following previous studies using composite scores to measure male involvement in pregnancy [13, 38]. The categorization was based on the distribution of the number of activities in which male partners participated. The low category consisted of zero shared decisions and zero ANC and birth preparedness activities, medium of 1 – 2 shared decisions and 1 – 3 ANC and birth preparedness activities, and high of 3 shared decisions and 4 – 7 ANC and birth preparedness activities.

#### Exploratory variables

##### Gender-equitable attitude

This variable was assessed using the gender equitable men’s (GEM) scale, which measures attitudes towards gender norms in intimate relationships or differing social expectations for men and women. Initially developed by Promundo and Program for Appropriate Technology in Health (PATH) for use with young Brazilian men [39], the scale has been adapted in different settings worldwide, including Ethiopia, China, India, Kenya, Tanzania, Uganda and the DRC [40, 41]. Although the number of items included varies in different country applications, the GEM scale is a sensitive and cross-culturally relevant scale with good predictive validity and Cronbach alphas range from 0.72 to 0.83 [40].

The baseline survey included 17 items on violence, sexual relationship, masculinity, and gender norms and relationships that were scored on a 3-point scale (totally agree, partially agree, and disagree). Several steps were taken to construct the GEM score. First, all the items were coded in the appropriate direction. High scores represented high support for gender-equitable norms and some items were reverse-coded if a high score reflected low support for gender equity. Secondly, item analysis and factor analysis were conducted. An oblique rotation was used in the factor analysis to allow some correlation among the factors [42]. Items loading less than 0.30 and with a negative correlation coefficient were dropped [42]. This resulted in a one-factor model (eigen value = 2.18) with a total variance of 80%. Then, the final items were summed to create an additive scale [43], and higher GEM scores indicated more equitable attitudes towards gender norms (α = 0.7221; range = 11 – 33). For the CFA model fit indices and the descriptive statistics of the individual components of the scale, see Table S1 and S2 in Additional File 1, respectively.

##### Knowledge of ANC, birth preparedness, and danger signs

Male partners were asked a series of questions to measure their knowledge of antenatal, danger signs, and birth preparedness.

- *ANC benefits*. To measure knowledge of the benefits of ANC, they were asked to mention three important benefits of a woman seeing someone for ANC when she is pregnant. The benefits were not read out loud; instead, all responses provided were recorded either by selecting options provided in the survey or entering the response if it was not listed. A summative score was constructed and categorized as knowledge of 0–1 benefits, two benefits and three or more benefits.
- *Timing of ANC visit*. Knowledge of the timing of ANC visits was measured by asking male partners, “in what month of pregnancy should a woman start attending antenatal care visits?” Responses were coded as (1) during the first trimester and (2) after the first trimester.
- *Number of ANC visits*. Male partners were asked “how many times should a pregnant woman go for antenatal care?” At the time of data collection, the DRC Ministry of Health had not updated its recommended number of ANC visits to comply with the 2016 WHO Guidelines on Antenatal Care for a Positive Pregnancy [44], which recommends 8 or more ANC visits. Consequently, we defined adequate ANC as four or more ANC visits.
- *Danger signs and birth preparedness*. Knowledge of danger signs for mother and newborn was assessed by asking male partners, “what danger signs during pregnancy, delivery or soon thereafter do you know that need immediate medical attention?” and “what signs tell you that your newborn is in danger and needs healthcare right away?” To measure their knowledge of birth preparedness, male partners were asked, “how can you and [name of first-time mother] prepare for a possible maternal emergency?” Three summative scores were created to measure a male partner’s knowledge of (a) danger signs for mother, (b) newborn danger signs and (c) birth preparedness steps. A higher score indicated greater knowledge of each construct being measured.

##### Self-efficacy

The generalized self-efficacy scale was used to measure a male partner’s self-efficacy. Male partners were asked about their level of agreement (not at all true, hardly true, moderately true, or exactly true) with the ten items in the scale. Items in the scale were summed up such that the higher values signified greater self-efficacy and capacity to execute behavior (α = 0.7573; range = 0 – 40). See Table S3 in Additional File 1 for the descriptive statistics of the individual components of the scale.

##### Co-parental relationship factors

Questions on relationship satisfaction, perceived power, and intimate partner violence were used to measure the co-parental relationship factors between FTM and their male partners.

- *Relationship satisfaction.* Male partners assessed their relationship with their FTM using the Relationship Assessment Scale (RAS). The 7-item scale was designed to measure an individual’s satisfaction with their relationship [45]. Items were scored on a 5-point Likert scale, ranging from 1 (low satisfaction) to 5 (high satisfaction). For instance, men were asked about how well does the FTM meet their needs and how much they loved their FTM. Factor analysis revealed a one-factor model (eigen value = 2.88), and the summation of the items resulted in a scale ranging from 7 to 35. Higher scores on the scale signified better relationship satisfaction. The reliability of the RAS in this study, α = 0. 7992, is comparable to previous studies that reported Cronbach alpha scores ranging from 0.80 – 0.91 [46, 47]. See Tables S1 and S4 in Additional File 1 for detailed information on descriptive statistics of the items in the scale and the CFA model fit indices.
- *Intimate partner violence (IPV).* Emotional, physical, and sexual IPV perpetration against the FTM was measured using an adapted DHS domestic violence module. The DHS module uses an abbreviated version of the Conflict Tactics Scale (CTS) [48] to measure women’s IPV victimization. Only male partners with adequate privacy during the interview were asked whether they had ever perpetrated a series of violent acts against the FTM. Those who responded in the affirmative to a particular item were then asked about the frequency with which they had perpetrated the violent behavior/act (often, sometimes, or not at all) in the 12 months preceding the interview. Male partners who answered “yes” to any of the items under the emotional, physical, or sexual violence subscale were considered perpetrators of each type of violence.

#### Socio-demographic variables

Socio-demographic variables were identified based on existing literature and the responsible fatherhood framework. They include the male partner’s age, marital status, education level, ethnicity, health zone, duration of living in the health zone, household wealth, number of children, employment, duration of employment, employment by both the male partner and FTM, and age difference between the male partner and FTM. The household wealth index, an asset index score, was constructed using principal component analysis (α = 0.6884). Households were ranked according to their use of improved drinking water sources, type of toilet, materials of the dwelling (floor, wall, and roofing), availability of electricity, and ownership of household items (radio, television, telephone, computer, refrigerator, stove, watch, mobile phone, bicycle, motorcycle, animal-drawn cart, car, and a boat with a motor). The index, made up of 19 items, was then divided into three tiers (low, middle, high). Table S5 in Additional File 1 presents a complete description of the variables that were used in the analysis.

### C. Analytical strategy

Frequencies, percentages, and means were presented to summarize the data. Bivariate analysis was used to describe the socio-demographic composition of the different levels of involvement. For this analysis, the significance between male involvement and independent variables was determined using Pearson’s chi-square test, Pearson correlation, and one-way analysis of variance (ANOVA), depending on the nature of the variables. For instance, Pearson’s chi square test was used to determine the relationship between the categorical exploratory variables and percentage of men who did not participate in any pregnancy-related activity and Pearson correlation coefficient was used to assess the relationship between two continuous variables, such as continuous exploratory variables and the outcome variables.

Linear regression (ordinary least squares [OLS]) was used to explore male involvement as a continuous variable. Guided by the conceptual framework in Fig. 1, all the exploratory variables of interest were included in the regression models for each outcome, while controlling for socio-demographic characteristics. Parameter estimates were used to evaluate the association between the outcome and exploratory variables. The second linear regression model was used to explore moderating effects where appropriate. Four two-way interaction terms were included between relationship satisfaction and the following a) gender-equitable attitude, b) physical violence, c) emotional violence, and d) sexual violence. The potential moderating effect of age was explored by performing a stratified analysis (15 – 24 years and 25 + years) on the first model. A three-way interaction term with age group would have been included in a third model if the estimates obtained had been significant and in opposite directions. None of the estimates in the stratified analysis had this issue; therefore, a third model was not included in the final analysis.

For each interaction term, graphical plots were created using the “*marginsplot*” command and “*margins*, *dydx()*” was used to obtain the marginal effect of the moderator. Additionally, the significance of the interaction terms was confirmed using the “*testparm*” command (test of joint significance). Multicollinearity among explanatory variables was detected using the variance inflation factor (VIF). The presence of multicollinearity could possibly lead to the inflation of the variance of parameter estimates. VIF less than four was used to demonstrate the absence of multicollinearity in the model [49]. For the ordinal measure of involvement, ordered logistic regression was not conducted because of the failure to meet the proportional odds assumption. All statistical analyses were carried out using STATA v.15 software [50], with statistical significance indicated by a *p*-value less than 0.05.

## Results

### Participant characteristics

Overall, most respondents were ever married (86%), worked for cash only (80%), and did not have children (73%; Table 2). Over two in five had completed secondary education (46%), lived continuously in the health zone for less than five years (43%), were 5–9 years older than the FTM (44%) and reported either Bas Kasai or Kwilu-Kwango as their ethnicity (42%). Half of the respondents worked throughout the year (52%) and only nine percent of male partners and FTMs received cash earnings. Two in five had perpetrated IPV in the past 12 months (40%). Physical IPV perpetration was the most prevalent form of IPV (33%). The prevalence rates for emotional and sexual IPV perpetration were 17% and 9%, respectively. Respondents had moderate levels of self-efficacy (mean = 34.3; SD = 4.6), high levels of relationship satisfaction (mean = 29.6; SD = 5.1), and moderate gender-equitable attitudes (GEM scale: mean = 21.6; SD = 4.8). The average age of male partners was 28 years old (SD = 5.90).

**Table 2:** Percentage distribution of the characteristics of male partners interviewed at baseline, Kinshasa 2018

Characteristics	n	Percentage (%)
**Average age (SD; range: 15—75)**	1,674	27.58 (5.90)
**Level of education**		
Lower than secondary	554	33.1
Secondary complete	769	45.9
Higher	351	21.0
**Marital** **Status**		
Never married	238	14.2
Ever married	1,436	85.8
**Ethnicity**		
Bakongo	497	29.7
Bas Kasai & Kwilu-Kwango	699	41.8
Kasai/Katanga /Tanganyika	221	13.2
Other	257	15.4
**Health zone of residence**		
Bumbu	206	12.3
Kingasani	387	23.1
Lemba	226	13.5
Masina1	373	22.3
Matete	180	10.8
Ndjili	302	18.0
**Duration of residence in the health zone**		
< 5 years	722	43.1
5 + years	303	18.1
Always	595	35.5
Visitor	54	3.2
**Number of children ever fathered**		
0	1,228	73.4
1	292	17.4
2 +	154	9.2
**Household wealth**		
Low	569	34.0
Middle	552	33.0
High	553	33.0
**Employment in the past 12 months**		
No Work	178	10.6
Work for cash only	1,341	80.1
Work but not paid, worked for kind or cash and kind	155	9.3
**Duration of employment**		
Unemployed	253	15.1
Throughout the year	872	52.1
Seasonally	241	14.4
Occasionally	308	18.4
**Dual employment**		
No	1,231	91.4
Yes	443	8.6
**Relative age difference between the FTM and MP**		
MP younger/ < 5 years older	494	29.5
5—9 years older	729	43.5
10 + years older	451	26.9
**Knowledge of ANC benefits**		
0—1	298	17.8
2	554	33.1
3 +	822	49.1
**Knowledge of the number of ANC visits**		
< 4 times	589	35.2
≥ 4 times	1,085	64.8
**Knowledge of the start of ANC**		
After first trimester	869	51.9
During first trimester	805	48.1
**Knowledge about danger signs for mother**		
0	123	6.2
1	515	22.6
2	536	37.6
3 +	500	33.6
**Knowledge about danger signs for newborns**		
0	103	7.3
1	379	30.8
2	629	32.0
3 +	563	29.9
**Knowledge of birth preparedness steps**		
0	92	5.5
1	944	56.4
2	523	31.2
3 +	115	6.9
**Past-year perpetuation of emotional violence**^**‡**^		
No	1,219	83.4
Yes	242	16.6
**Past-year perpetuation of physical violence**^**‡**^		
No	974	66.7
Yes	487	33.3
**Past-year perpetuation of sexual violence**^**‡**^		
No	1,336	91.4
Yes	125	8.6
**Total**	**1,674**	**100.0**
		*Mean (SD)*
**Relationship satisfaction (range: 7—35)**	1,674	29.62 (5.06)
**Gender-equitable attitude (range: 11—33)**	1,674	21.63 (4.83)
**Perceived self-efficacy (range: 13—40)**	1,674	34.16 (4.61)

*SD* Standard deviation, *FTM* fist-time mother, *MP* male partner

^**‡**^Only men who had privacy during the interview were asked IPV questions (15–24 years (*N* = 484); 25 + years (*N* = 977); 15 + years (*N* = 1,461)

For some categorical variables, column totals may not add up to 100 due to rounding

Knowledge of danger signs and birth preparedness was low. About a third of male partners knew three or more danger signs for the mother during pregnancy, delivery or soon thereafter that need immediate medical attention (34%), three in ten knew three or more danger signs for newborns (30%), and under one in ten knew three or more ways to prepare for a possible maternal emergency (7%). Male partners’ knowledge of ANC was slightly higher than their knowledge of danger signs and birth preparedness. Over three in five reported that FTMs must have four or more ANC visits (65%), about half knew three or more ANC benefits (49%), and reported FTMs must start ANC in the first trimester (48%).

### Participation in pregnancy-related activities

As shown in Table 3, male involvement in individual pregnancy-related activities during the first six months of pregnancy was relatively low. Less than half of male partners reported participating in saving for medical emergencies (49%), making decisions about ANC (43%), making a birth plan (40%), and arranging transportation for delivery (36%; Table 3). Only a third made decisions with the FTM about when and where to seek care and treatment for danger signs (33% and 29%, respectively), and about 21% made shared decisions about where to deliver the baby. About a quarter participated in finding information about pregnancy (26%) and under one in five were present at an ANC visit (19%) and participated in deciding on skilled attendance at delivery (19%). Participation in finding a blood donor had the lowest participation, only one in ten participated in this activity (11%).

**Table 3:** Percentage of male partners who were participated in pregnancy related activities, by age group, Kinshasa 2018

Pregnancy-related activities	n	Percentage (%)	N
***Antenatal care & birth preparedness***			
Present at antenatal care visit	280	19.1	1,469
Participated in finding information about the pregnancy	437	26.1	1,674
Participated in making decisions about antenatal care	716	42.8	1,674
Participated in making a birth plan	675	40.3	1,674
Participated in saving money for emergencies	821	49.0	1,674
Participated in arranging transport for delivery	595	35.5	1,674
Participated in deciding on skilled attendance at delivery	314	18.8	1,674
Participated in finding a blood donor	179	10.7	1,674
* Participation in no ANC & birth preparedness activity†*	715	42.7	1,674
* Participation in one ANC & birth preparedness activity†*	959	57.3	1,674
* Participation in all ANC & birth preparedness activities†*	55	3.3	1,674
***Shared decisions***			
Joint participation in deciding where to deliver the baby	356	21.3	1,674
Joint participation in deciding when to seek care and treatment for danger signs	531	32.7	1,674
Joint participation in deciding where to seek care and treatment for danger signs	480	28.7	1,674
* Participation in no shared decision*	941	56.2	1,674
* Participation in one shared decision*	733	43.8	1,674
* Participation in all shared decisions*	196	11.7	1,674

Median participation for antenatal care and birth preparedness is 2 (interquartile range (IQR) = 4), and median participation for shared decisions is 0 (IQR = 2)

^†^Excludes male partners’ presence at antenatal care visits because it was not included in the overall male involvement score

About half of the male partners participated in at least one ANC and birth preparedness activity and less than five percent participated in all seven activities included in the male involvement score. Participation in shared decisions followed a similar pattern, with 44% participating in at least one decision and 12% participating in all three decisions.

### Bivariate results

Table 4 provide the bivariate relationships between each predictor variable and the male involvement outcomes. Male involvement is presented as a score, where higher scores are indicative of higher participation in ANC and birth preparedness and shared decisions. In Table S7 in Additional File 1, male involvement is presented as an ordered variable, where the low category consists of zero shared decisions and zero ANC and birth preparedness activities, medium of 1 – 2 shared decisions and 1 – 3 ANC and birth preparedness activities, and high of 3 shared decisions and 4 – 7 ANC and birth preparedness activities.

**Table 4:** Percentage of male partners involved in no decisions and mean number of antenatal care and birth preparedness activities and shared decisions that male partners are involved in, by male partners' knowledge, co-parental relationship, self-efficacy, and gender-equitable attitudes, Kinshasa 2018

	**ANC and birth preparedness**			**Shared decisions**		
**Independent Variables**	**% participating in no activity**	**Mean # of activities (SD)**		**% participating in no activity**	**Mean # of activities (SD)**	
**Knowledge of ANC benefits**			***			***
0 – 1	51.3	1.72 (2.09)		64.8	0.57 (0.88)	
2	37.0	2.39 (2.23)		56.9	0.81 (1.06)	
3 +	43.4	2.31 (2.42)		52.7	0.91 (1.12)	
**Knowledge of the number of ANC visits**			***			*
< 4 times	50.6	1.73 (2.10)		58.4	0.73 (0.99)	
≥ 4 times	38.4	2.50 (2.38)		55.0	0.86 (1.10)	
**Knowledge of the start of ANC**						
After first trimester	41.8	2.22 (2.27)		57.2	0.78 (1.05)	
During first trimester	43.7	2.25 (2.36)		55.2	0.85 (1.09)	
**Knowledge about danger signs for mother**			***			***
0^†^	70.9	1.17 (2.01)		68.0	0.57 (0.95)	
1	38.0	2.35 (2.23)		61.7	0.63 (0.92)	
2	44.8	2.16 (2.31)		56.1	0.82 (1.06)	
3 +	38.4	2.44 (2.37)		50.4	0.98 (1.15)	
**Knowledge about danger signs for newborns**			***			***
0^†^	64.2	1.37 (2.09)		61.8	0.59 (0.85)	
1	43.1	2.23 (2.30)		62.3	0.70 (1.04)	
2	37.5	2.40 (2.28)		53.9	0.86 (1.08)	
3 +	42.6	2.28 (2.37)		51.0	0.94 (1.12)	
**Knowledge of birth preparedness steps**			***			***
0^†^	71.7	0.73 (1.47)		67.4	0.55 (0.92)	
1	48.6	1.83 (2.12)		59.1	0.73 (1.01)	
2	30.2	3.01 (2.41)		52.8	0.94 (1.15)	
3 +	27.8	3.19 (2.42)		39.1	1.16 (1.14)	
**Past-year perpetuation of emotional violence**^‡^						***
No	44.2	2.20 (2.33)		53.5	0.89 (1.10)	
Yes	41.7	2.37 (2.40)		64.9	0.60 (0.92)	
**Past-year perpetuation of physical violence**^‡^			*			**
No	41.4	2.33 (2.35)		53.7	0.90 (1.12)	
Yes	48.7	2.02 (2.32)		58.7	0.72 (0.98)	
**Past-year perpetuation of sexual violence**^‡^						*
No	43.4	2.25 (2.35)		54.7	0.86 (1.09)	
Yes	48.0	2.01 (2.25)		62.4	0.63 (0.95)	
**Total**	**42.7**	**2.23 (2.34)**		**55.7**	**0.84 (1.08)**	
	*Mean (SD)*	*Rho (p)*		*Mean (SD)*	*Rho (p)*	
**Relationship satisfaction (range: 7—35)**	29.62 (5.49)	0.07	**	29.34 (5.23)	0.05	
**Gender-equitable attitude (range: 11—33)**	21.86 (4.97)	-0.04		21.08 (4.76)	0.16	***
**Perceived self-efficacy (range: 13—40)**	33.64 (5.22)	0.20	***	34.59 (4.54)	-0.06	*
N	1,674					

*ANC* antenatal care, *IPV* intimate partner violence, *SD* Standard deviation

^†^The number of partners with low, medium, and high involvement in at least one cell in the category was less than 25

^‡^Only men who had privacy during the interview were asked IPV questions (*N* = 1,461)

^***^< 0.001; ** < 0.01; * < 0.05

#### Involvement in ANC and birth preparedness

Male involvement in ANC and birth preparedness activities was low (Table 4). On average, male partners participated in two activities (mean = 2.2; SD = 2.3) and over two in five did not participate in any ANC and birth preparedness activity. As shown in Table S7 in Additional File 1, about a quarter of male partners had medium level of involvement (24%, participation in one to three activities) and a third had high level of involvement (33%, participation in 4 to 7 activities).

Significant variation was seen in the average involvement in ANC and birth preparedness activities across the levels of knowledge of ANC benefits, number of ANC visits, birth preparedness steps, and danger signs for mothers and newborns. Male partners who mentioned that FTMs should have four or more ANC visits participated in significantly more activities than those who reported less than four visits (mean = 2.5 versus mean = 1.7 activities). Significantly more male partners who had not perpetrated physical IPV had higher involvement compared to those who had perpetrated physical IPV (mean = 2.3 versus mean = 2.0).

Involvement was positively correlated with relationship satisfaction (*r* = 0.07) and perceived self-efficacy (*r* = 0.20). These correlations were statistically significant, and similar associations were seen for the level of involvement (see Table S7 in Additional File 1). For instance, male partners with higher involvement, participating in 4 – 7 activities, had better relationship satisfaction (mean = 30.2; SD = 5.1) and self-efficacy (mean = 35.5; SD = 3.7) compared to those with lower levels of involvement.

#### Involvement in shared decisions

Involvement in shared decisions was also low (Table 4), with male partners participation in an average of 0.8 decisions (SD = 1.1). Over half of the male partners (56%) had low involvement, followed by high involvement (27%) and medium involvement (18%; see Table S7 in Additional File 1).

Although the absolute difference was small, male partners who did not perpetrate any form of IPV participated in significantly more shared decisions (mean = 0.9, SD = 1.1, not shown) than those who did (mean = 0.7, SD = 1.1, *p* < 0.01; not shown in Table 4). For the individual types of violence, involvement was also significantly higher among non-perpetrators of IPV than among perpetrators. Emotional IPV perpetrators had the lowest participation (mean = 0.6, SD = 0.92) compared to physical IPV perpetrators (mean = 0.72, SD = 0.98) and sexual IPV perpetrators (mean = 0.63, SD = 0.95).

Knowledge of ANC benefits, danger signs, and birth preparedness were positively associated with higher involvement in shared decisions. For instance, male partners with knowledge of three or more danger signs for newborns were highly involved in shared decisions compared to those with knowledge of one danger sign (mean = 0.94 versus mean = 0.70). Contrary to involvement in ANC and birth preparedness, self-efficacy was negatively correlated with involvement in shared decisions (*r* = -0.06, *p* < 0.05, Table 4) and was highest among male partners with low involvement (mean = 34.5; SD = 4.5, Table S7). Gender-equitable attitude was positively correlated with involvement (*r* = 0.16, *p* < 0.001, Table 4) and highest among those with high level of involvement in shared decisions (mean = 22.7; SD = 5.0, Table S7).

### Multivariate analysis results

#### Predictors of male partner involvement

Tables 5 and 6 present the multiple linear regression results for male involvement in ANC and birth preparedness and shared decisions after adjusting for socio-demographic characteristics. The findings suggest that different factors influence participation in ANC and birth preparedness and shared decisions (see Tables S8 and S9 in Additional File 1 for the full regression results, including the socio-demographic characteristics).

**Table 5:** Results of adjusted regression models of male involvement in antenatal care and birth preparedness, Kinshasa 2018

	**Male involvement in ANC and birth preparedness**					
	**Total sample**			**Total sample with interaction**		
**Independent Variables**	**Beta**		**95% CI**	**Beta**		**95% CI**
**Knowledge of ANC benefits**						
0 – 1	[REF]			[REF]		
2	0.192		[-0.142, 0.526]	0.187		[-0.147, 0.521]
3 +	0.04		[-0.306, 0.385]	0.052		[-0.294, 0.399]
**Knowledge of the number of ANC visits**						
< 4 times	[REF]			[REF]		
≥ 4 times	0.5222	**	[0.277, 0.767]	0.523	***	[0.278, 0.768]
**Knowledge of the start of ANC**						
After first trimester	[REF]			[REF]		
During first trimester	-0.077		[-0.298, 0.144]	-0.081		[-0.302, 0.140]
**Knowledge about danger signs for mother**						
0	[REF]			[REF]		
1	0.49		[-0.023, 1.003]	0.467		[-0.046, 0.980]
2	0.101		[-0.405, 0.606]	0.092		[-0.413, 0.597]
3 +	0.22		[-0.319, 0.760]	0.226		[-0.314, 0.765]
**Knowledge about danger signs for newborns**						
0	[REF]			[REF]		
1	0.462	*	[0.004, 0.919]	0.469		[0.009, 0.929]
2	0.415		[-0.063, 0.892]	0.436		[-0.042, 0.914]
3 +	0.159		[-0.359, 0.678]	0.191		[-0.328, 0.710]
**Knowledge of birth preparedness steps**						
0	[REF]			[REF]		
1	0.737	**	[0.215, 1.260]	0.732	**	[0.210, 1.254]
2	1.964	***	[1.408, 2.520]	1.957	***	[1.402, 2.513]
3 +	2.495	***	[1.812, 3.179]	2.464	***	[1.779, 3.149]
**Past-year perpetuation of emotional violence**						
No	[REF]			[REF]		
Yes	0.322		[-0.029, 0.673]	-0.643		[-2.469, 1.183]
**Past-year perpetuation of physical violence**						
No	[REF]			[REF]		
Yes	-0.195		[-0.471, 0.080]	-0.391		[-2.071, 1.288]
**Past-year perpetuation of sexual violence**						
No	[REF]			[REF]		
Yes	-0.133		[-0.546, 0.281]	-0.295		[-2.421, 1.831]
Relationship satisfaction	0.036	**	[0.012, 0.059]	-0.11	*	[-0.215, -0.004]
Gender-equitable attitude	-0.023		[-0.049, 0.002]	-0.222	**	[-0.368, -0.075]
Perceived self-efficacy	0.092	***	[0.066, 0.118]	0.093	***	[0.066, 0.119]
*Interaction terms*						
**Relationship satisfaction x gender-equitable attitude**				0.007	**	[0.002, 0.011]
**Relationship satisfaction x emotional IPV perpetration**				0.034		[-0.028, 0.097]
**Relationship satisfaction x physical IPV perpetration**				0.006		[-0.049, 0.061]
**Relationship satisfaction x sexual IPV perpetration**				0.006		[-0.069, 0.080]
Constant	-3.543	***	[-5.083, -2.004]	0.078		[-2.675, 4.238]
N	1,461			1,461		
adjusted R-squared	0.24			0.245		
VIF	1.26					

Regression models control for background variables including age, level of education, marital status, ethnicity, health zone of residence, duration of residence in the health zone, number of children fathered, household wealth, employment in the past 12 months, duration of employment, employment by both partners, and the relative age difference between the male partner and the first-time mother

*ANC *antenatal care,* Beta *unstandardized adjusted coefficient,* SE *Standard Error,* CI *confidence interval,* IPV *intimate partner violence,* REF* reference

^***^< 0.001; **< 0.01; *< 0.05

**Table 6:** Results of regression models of male involvement in shared decisions about pregnancy, Kinshasa 2018

	**Male involvement in shared decisions**					
	**Total sample**			**Total sample with interaction**		
**Independent Variables**	**Beta**		**95% CI**	**Beta**		**95% CI**
**Knowledge of ANC benefits**						
0 – 1	[REF]			[REF]		
2	0.195		[0.028, 0.363]	0.200	*	[0.032, 0.367]
3 +	0.218	*	[0.045, 0.391]	0.231	**	[0.058, 0.405]
**Knowledge of the number of ANC visits**						
< 4 times	[REF]			[REF]		
≥ 4 times	0.097		[-0.026, 0.219]	0.094		[-0.029, 0.217]
**Knowledge of the start of ANC**						
After first trimester	[REF]			[REF]		
During first trimester	-0.013		[-0.124, 0.098]	-0.014		[-0.125, 0.097]
**Knowledge about danger signs for mother**						
0	[REF]			[REF]		
1	-0.017		[-0.274, 0.240]	-0.016		[-0.273, 0.241]
2	0.127		[-0.126, 0.381]	0.132		[-0.121, 0.385]
3 +	0.227		[-0.043, 0.497]	0.238		[-0.033, 0.508]
**Knowledge about danger signs for newborns**						
0	[REF]			[REF]		
1	0.089		[-0.140, 0.318]	0.072		[-0.159, 0.303]
2	0.099		[-0.140, 0.338]	0.089		[-0.151, 0.329]
3 +	0.010		[-0.250, 0.269]	0.006		[-0.255, 0.266]
**Knowledge of birth preparedness steps**						
0	[REF]			[REF]		
1	0.040		[-0.221, 0.302]	0.032		[-0.229, 0.294]
2	0.165		[-0.113, 0.444]	0.159		[-0.119, 0.438]
3 +	0.275		[-0.067, 0.618]	0.253		[-0.091, 0.596]
**Past-year perpetuation of emotional violence**						
No	[REF]			[REF]		
Yes	-0.148		[-0.324, 0.028]	0.104		[-0.811, 1.019]
**Past-year perpetuation of physical violence**						
No	[REF]			[REF]		
Yes	-0.044		[-0.182, 0.094]	-0.175		[-1.017, 0.667]
**Past-year perpetuation of sexual violence**						
No	[REF]			[REF]		
Yes	0.025		[-0.182, 0.232]	-1.023		[-2.089, 0.042]
Relationship satisfaction	0.001		[-0.011, 0.012]	-0.054	*	[-0.107, -0.001]
Gender-equitable attitude	0.034	***	[0.021, 0.047]	-0.041		[-0.115, 0.032]
Perceived self-efficacy	-0.016	*	[-0.029, -0.002]	-0.016	*	[-0.029, -0.003]
*Interaction terms*						
**Relationship satisfaction x gender-equitable attitude**				0.002	*	[0.000, 0.005]
**Relationship satisfaction x emotional IPV perpetration**				-0.009		[-0.040, 0.023]
**Relationship satisfaction x physical IPV perpetration**				0.004		[-0.023, 0.032]
**Relationship satisfaction x sexual IPV perpetration**				0.037	*	[0.000, 0.075]
Constant	0.113		[-0.658, 0.885]	1.777	*	[0.045, 3.509]
N	1,461			1,461		
adjusted R-squared	0.10			0.11		
VIF	1.26					

Regression models control for background variables including age, level of education, marital status, ethnicity, health zone of residence, duration of residence in the health zone, number of children fathered, household wealth, employment in the past 12 months, duration of employment, employment by both partners, and the relative age difference between the male partner and the first-time mother

*ANC* antenatal care, *Beta* unstandardized adjusted coefficient, *SE* Standard Error, *CI* confidence interval, *IPV* intimate partner violence, *REF *reference

^***^< 0.001; **< 0.01; *< 0.05

#### Involvement in ANC and birth preparedness

After controlling for socio-demographic characteristics, the analysis revealed that knowledge of the DRC recommended number of ANC visits (β = 0.52, *p* < 0.01), knowledge of one or more birth preparedness steps (1: [β = 0.74, *p* < 0.01]; 2: [β = 1.96, *p* < 0.001]; 3 + : [β = 2.50, *p* < 0.001]), and knowledge of one newborn danger sign (β = 0.46, *p* < 0.05) were significantly associated with male involvement in ANC and birth preparedness. Of co-parental relationship factors, relationship satisfaction was the only significant predictor, regardless of age group. With each unit increase in a male partner’s relationship satisfaction, his involvement increased (Total: [β = 0.04, *p* < 0.01]; and 15–24 years: [β = 0.05, *p* < 0.05]; 25 + years: [β = 0.04, p < 0.05], not shown). Emotional IPV perpetration was a significant positive predictor of involvement for only older male partners (β = 0.64, *p* < 0.01, results not shown).

Self-efficacy was a significant predictor of involvement for the overall sample (β = 0.09, *p* < 0.001), as well as both age groups (15–24 years: [β = 0.10, *p* < 0.001]; 25 + years: [β = 0.09, *p* < 0.001], results not shown). Interestingly, gender-equitable attitudes had a negative, though statistically insignificant, association with involvement (β = -0.02, *p* > 0.05). For older male partners, this negative association was significant (β = -0.04, *p* < 0.05) such that their involvement decreases as gender-equitable attitude increases (results not shown).

Regarding the effects of the other variables included in the model, Table S8 in Additional File 1 shows that living in certain health zones (Lemba and Ndjili) was a predictor of involvement. Male partners who always lived in the health zone of residence (β = -0.29, *p* < 0.05) participated in fewer activities than male partners who lived in the health zone for less than five years. Compared to unemployed male partners, those working throughout the year (β = -0.90, *p* < 0.05), seasonally (β = -1.33, *p* < 0.001), and occasionally (β = -1.08, *p* < 0.01) were less involved. The duration of employment, specifically working seasonally and occasionally, was also significant for younger male partners (not shown).

#### Involvement in shared decisions

For shared decision-making, knowledge of two or more ANC benefits, gender-equitable attitudes, and self-efficacy were significant predictors (Table 6). Male partners who knew two or more ANC benefits participated in more activities than their counterparts who knew no benefits (2: [β = 0.20, *p* < 0.05]; 3 + : [β = 0.22, *p* < 0.05]). Converse to involvement in ANC and birth preparedness, gender-equitable attitudes (β = 0.03, *p* < 0.001) was a positive predictor and self-efficacy (β = -0.02, *p* < 0.05) was a negative predictor, such that more gender-equitable attitudes were associated with more shared decisions and greater self-efficacy was associated with fewer shared decisions. When disaggregated by age, the association between gender-equitable attitudes and shared decision making was significant for both age groups (15–24 years: [β = 0.04, *p* < 0.01]; 25 + years: [β = 0.03, *p* < 0.001], not shown), and self-efficacy was significant for the older male partners (25 + years: β = -0.02, *p* < 0.05, not shown).

The regression results for the socio-demographic characteristics presented in Table S9 in Additional File 1 show that male partners in a relationship with an employed FTM participated in more activities than their counterparts with an unemployed FTM (β = 0.14, *p* < 0.05). This was also observed for older male partners (β = 0.19, *p* < 0.05). Although the health zone of residence was not a significant predictor in the regression model for the total population, the age stratification analysis revealed that always living in the health zone of residence (β = 0.23, *p* < 0.05) was a positive predictor of involvement among younger male partners. The associations between involvement and the remaining socio-demographic characteristics (age group, level of education, marital status, ethnicity, duration of residence in the health zone, number of children fathered, household wealth, employment history, duration of employment, and age difference between male partner and FTM) were not statistically significant (*p* > 0.05).

### Moderation analysis results

The third research objective aimed to answer the question, “to what extent do gender equitable attitudes or IPV perpetration moderate the association between relationship satisfaction and male involvement during pregnancy?” by introducing interaction terms into the regression model for each outcome. During the preliminary analysis, each interaction term was added to the first model before the inclusion of all terms in the final model presented. The tests of joint significance of the interaction terms in the final model (including all the terms) were significant (*p* = 0.008 for ANC and birth planning and *p* < 0.001 for shared decisions). The regression results of the moderation analysis are presented in Tables 5 and 6, and the average marginal effects of the moderators as are presented in Table 8. The average marginal effect is the predicted change in one group compared to the reference group, assuming all other covariates are constant.

#### Involvement in ANC and birth preparedness

**Fig. 2:**
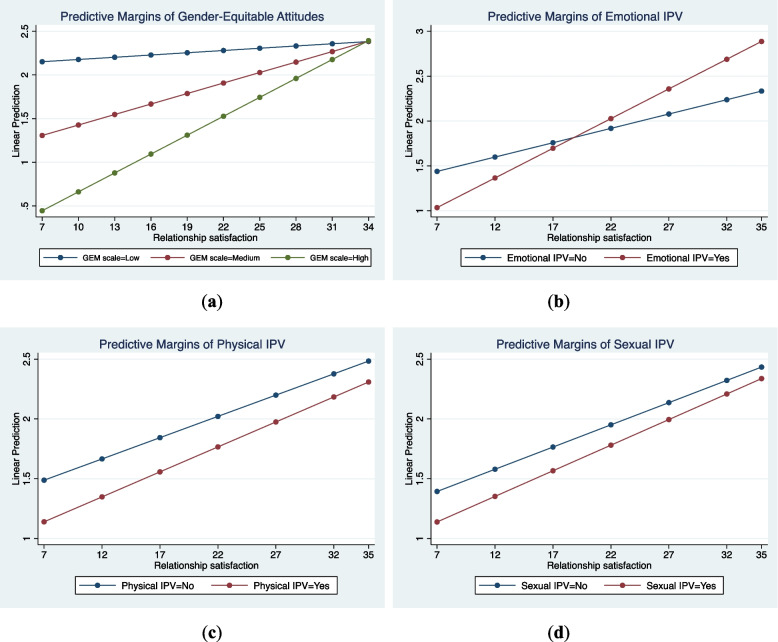
Plots of the predicted margins of the moderators (**A**: gender-equitable attitude; **B**: emotional intimate partner violence; **C**: physical intimate partner violence; **D**: sexual intimate partner violence) in the relationship between relationship satisfaction and involvement in antenatal care and birth preparedness activities

Gender-equitable attitude was a significant moderator of the association between relationship satisfaction and involvement in ANC and birth preparedness (β = 0.01, *p* < 0.01). Examination of the marginal plot (shown in Fig. 2A) and the average marginal effect in Table 7 confirms our hypothesis; as gender-equitable attitudes increased, the positive effect of relationship satisfaction on involvement in ANC and birth preparedness increased. Relationship satisfaction had the highest positive effect for male partners categorized as having a high GEM scale. For these men, the probability of participation increased by seven percentage points for each unit increase in relationship satisfaction (average marginal effect = 0.07, *p* < 0.001, Table 7). This was followed by those with medium/moderate gender-equitable attitudes (average marginal effect = 0.04, *p* < 0.01).

**Table 7:** Average marginal effects of the moderators in the relationship between relationship satisfaction and male involvement in shared decision-making and antenatal care and birth preparedness, Kinshasa 2018

	**Male involvement in ANC and birth preparedness**				**Male involvement in shared decisions**			
	**Average Marginal Effect**				**Average Marginal Effect**			
**Moderators**	**Each Level**	***p*-value**	**Relative to Reference**	***p*-value**	**Each Level**	***p*-value**	**Relative to Reference**	***p*-value**
**RS x Emotional IPV**								
No	0.032	0.023	[REF]		0.003	0.631	[REF]	
Yes	0.066	0.018	0.034	0.284	-0.005	0.700	-0.009	0.560
**RS x Physical IPV**								
No	0.036	0.022	[REF]		0.001	0.946	[REF]	
Yes	0.042	0.063	0.006	0.825	0.005	0.671	0.004	0.763
**RS x Sexual IPV**								
No	0.037	0.004	[REF]		-0.001	0.846	[REF]	
Yes	0.043	0.242	0.006	0.881	0.036	0.049	0.037	0.049
**RS x Gender-equitable attitude**								
Low	0.009	0.577	[REF]		-0.009	0.241	[REF]	
Medium	0.040	0.001	0.031	0.007	0.003	0.649	0.012	0.043
High	0.072	0.000	0.064	0.007	0.015	0.113	0.024	0.043

*ANC* antenatal care, *IPV* intimate partner violence, *REF* reference group, *RS* relationship satisfaction

Figures 2B, C, and D show the plots of predicted margins of emotional, physical, and sexual IPV, respectively. The regression results indicated that none of these moderators were significant, although Fig. (2B) suggested that emotional violence could moderate the relationship of relationship satisfaction with involvement.

#### Involvement in shared decisions

**Fig. 3:**
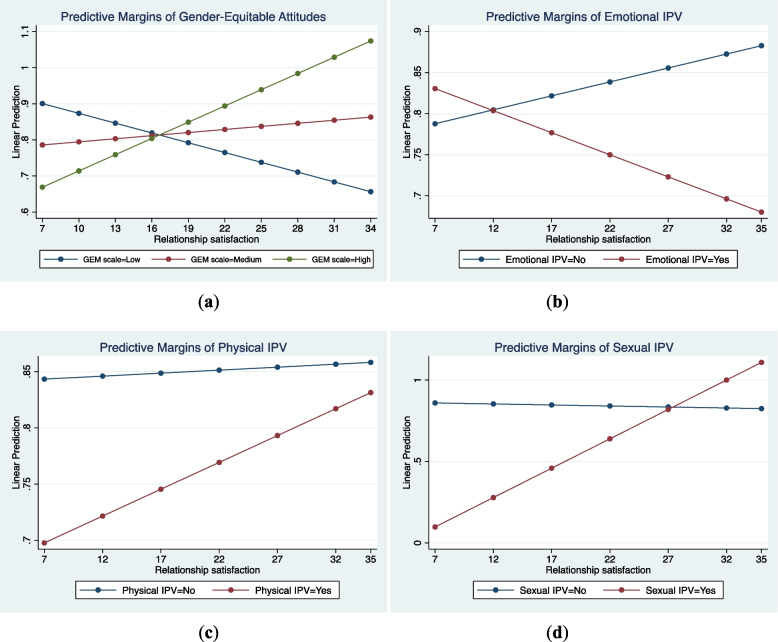
Plots of the predicted margins of the moderators (**A**: gender-equitable attitude; **B**: emotional intimate partner violence; **C**: physical intimate partner violence; **D**: sexual intimate partner violence) in the relationship between relationship satisfaction and involvement in shared decisions

As shown in Tables 6 and 7, emotional and physical violence were not significant moderators of the association between relationship satisfaction and participation in shared decisions (*p* > 0.05; see Figs. 3B and C). However, gender-equitable attitudes and sexual IPV perpetration were significant moderators in this relationship, as suggested by their significant interaction terms (β = 0.002, *p* < 0.05; β = 0.04, *p* < 0.05, respectively). Contrary to our expectations, sexual IPV perpetration had a positive effect on involvement in shared decisions. As relationship satisfaction increased, shared decision-making among male partners who perpetrated sexual IPV increased by 3.7 percentage points compared to those who did not (average marginal effect = 0.037, *p* = 0.04, Table 8). Results further suggest that increasing relationship satisfaction had a greater effect among men who perpetrated sexual IPV (Fig. 3D). For gender-equitable attitudes, the results supported our hypothesis. Similar to involvement in ANC and birth preparedness, having medium and high relative to low gender-equitable attitudes increased the probability of shared decisions (average marginal effect = 0.012 and 0.024, respectively). Also, the positive effect of relationship satisfaction on involvement in shared decisions was greatest for male partners with high gender-equitable attitudes (Fig. 3A).

## Discussion

This analysis examined the patterns and predictors of male involvement during pregnancy. Male partner involvement in ANC and birth preparedness and shared decision-making was low, with male partners participating in an average of two ANC and birth preparedness activities (out of 7) and one pregnancy-related decision (out of 3). Only a third had high levels of involvement in ANC and birth preparedness activities and 27% had high levels of shared decisions. For the specific activities, saving for a medical emergency had the highest level of involvement (49%), while finding a blood donor had the lowest (11%). As expected, knowledge was positively associated with involvement. Male partners who knew that a woman should attend four or more ANC visits, knew one newborn danger sign, and knew more than one birth preparedness step were more involved in ANC and birth preparedness. In contrast, for shared decisions, male partners who knew two or more ANC benefits were more involved than those who knew one or no benefit. Relationship satisfaction was positively associated with involvement in ANC and birth preparedness and male partners with higher gender-equitable attitudes were more involved in shared decisions. Self-efficacy was a positive predictor of involvement in ANC and birth preparedness but a negative predictor of shared decisions.

These findings contribute to the male involvement literature and although studies have used different approaches for measuring involvement, the low levels of involvement among male partners in the present study are consistent with previous findings from other sub-Saharan countries [13, 38, 51]. In Kenya, 19% of men had high male involvement (participation in 3–5 activities) [38], 20% who participated in three to four maternity care activities in Tanzania had high involvement [52], and in Uganda, 26% of men whose wives attended ANC had high involvement scores (participation in 4–6 activities) [51]. Also, in Kenya, Hampanda et al. [13] found that men actively participated in 1.4 activities. However, a few studies have found higher estimates of male involvement. A quasi-experimental study in Tanzania found that about two in five men participated in at least three ANC and birth preparedness activities at baseline and this increased to 81% at endline; similarly shared decision-making increased from 47 to 87% [11]. Another study in Ethiopia found that three in five men saved money for emergencies (63%), and a lower percentage participated in identifying a blood donor (12%) [53]. It’s worth noting that the low prevalence of involvement is consistent with studies in the DRC, even though these studies used binary measures [16].

In line with the findings, studies in the DRC and sub-Saharan Africa found gender-equitable attitudes [19, 54], strong relationships between the couple [22, 55, 56], and maternal and child health knowledge were positive predictors of involvement. Studies exploring the association of involvement with knowledge used various indicators to measure knowledge [38, 57]; despite this, our findings were similar to theirs. For instance, men with knowledge of ANC services in Ethiopia were five times as likely to be involved [57], and male partners who had read the mother–child booklet after ANC visits were twice as likely to be involved [38]. In our study, emotional, sexual, and physical IPV perpetration did not significantly hinder any form of involvement. However, among the older male partners involvement, perpetrators of emotional violence were more involved in ANC and birth preparedness than non-perpetrators. This is not consistent with findings that suggest that IPV hinders involvement [22, 55]. Often a precursor to physical IPV, emotional IPV includes verbal abuse, dominance, isolation, ridicule and targets the victim’s phycological well-being. Thus, older male partners could potentially use their involvement as a way to isolate the FTM further and perpetrate emotional IPV. However, this cannot be ascertained because our study is cross-sectional.

Interestingly, self-efficacy did not uniformly influence involvement and the association differed depending on the outcome. Its positive association with ANC and birth preparedness was consistent with other findings [58], while the inverse relationship observed for shared decisions diverged from previous findings. This result implies that increasing a person’s belief in their ability to execute a behavior does not always lead to them performing the behavior. In organizational research, researchers argue that perceptions of self-efficacy are not formed in a vacuum but are influenced by contextual factors and the characteristics of the activity [59, 60]. Within the context of this study, it is possible that male partners’ perception of the task's complexity and significance could influence their self-efficacy and, thereby, their involvement. To better understand our findings, qualitative research should be conducted to understand the contextual factors at play that may influence self-efficacy, and future research should use a scale that specifically measures parental self-efficacy. The study revealed that socio-demographic factors, including health zone of residence, duration of residence in the health zone, and duration of employment, are important for male involvement in ANC and birth preparedness, supporting the findings from previous studies [61, 62]. Employment of both partners encouraged male involvement in shared decisions [62, 63].

The findings also suggested that gender-equitable attitudes and sexual IPV were significant moderators, but the latter result was not in the expected direction. For both forms of involvement, having higher gender-equitable attitudes increased the association between relationship satisfaction and involvement, while involvement in shared decisions increased with each unit increase in relationship satisfaction for sexual IPV perpetrators. This unexpected finding highlights that in the midst of sexual IPV perpetration, the male partner can be involved if he is satisfied with his relationship. However, in doing so, it could further promote IPV and thus, unequal gender power relations.

These findings have implications for programs seeking to improve male involvement to ultimately address gender-based health inequities. The moderating effect of gender-equitable attitudes emphasizes the need for programs to be intentional about sensitizing male partners, especially older male partners, to dispel attitudes that promote unequal gender power relations and inequities. Programs should take into account the context and the strategies used to improve male involvement should not be done at the expense of the woman. Programs should also embed activities that address multiple determinants of male involvement in shared decisions and ANC and birth preparedness. For instance, given that the male partner’s satisfaction with his relationships with the FTM matters in his decision to be involved, there is the need to promote activities that promote couple communication, reduce IPV, and consequently improve relationship quality [64]. The interventions should also increase knowledge of various aspects of maternal and child health as knowledge was an important predictor. Although this study did not assess the impact of multiple approaches, interventions should follow the WHO recommendation and consider how to incorporate multiple approaches that address the above mentioned factors to increase their effectiveness [64]. Lastly, programs should use more comprehensive measures to assess male involvement during monitoring and evaluation as the concept is nuanced and cannot be fully captured with a single indicator. This is important from a monitoring and evaluation standpoint, but involvement also varies depending on the type of male involvement; thus, it is important to acknowledge and incorporate this variation into the program’s approach.

### Strengths and limitations

Most studies within sub-Saharan Africa have studied male involvement in the various stages of pregnancy individually and have conceptualized the term as a binary variable. This study provides a more comprehensive definition by including several pregnancy-related activities in one measure. Secondly, most studies in the DRC have studied male involvement as part of a larger study focused on HIV, and the present study focuses on male involvement in pregnancy outside the realm of HIV. Furthermore, we shed light on the association between attitudes toward gender norms, knowledge of antenatal care and birth preparedness, intimate partner violence, mass media, socio-demographic factors, and male involvement in pregnancy.

Several limitations are also recognized. Since this is cross-sectional data, it is difficult to establish causality or temporal ordering. Studying male involvement with longitudinal data on men’s behavior during consecutive births may give us a better insight into the factors that encourage male involvement. Secondly, the measures of male involvement and possible predictors are based on self-report, which could be affected by social desirability or recall bias. Regarding the recruitment of study participants, not all male partners of FTMs were recruited and enrolled in the study. FTMs had to consent to male partner participation before male partners could be contacted. Not all FTMs consented to have their male partners contacted, and not all male partners consented to be in the study. There were also 305 male partners of FTMs (17%) who were interviewed but were not included in the sample analyzed in the regression models. Male partners included and those not included in the analysis were not statistically different for most variables, thus are comparable on observed factors. Although many predictors of male involvement were measured in the baseline survey, it did not include measures that previous research found to be associated with male involvement (e.g., number of wives, health facility factors, social support, gender of the child (the baby was not yet born, except if they did an ultrasound to find out the baby’s sex), and previous involvement of male partner’s own father).

Additionally, the baseline study was conducted when the FTM was approximately six months pregnant; therefore, the measure of male partner involvement represents a truncated experience. This could bias the estimate obtained for male involvement because male partners could have become involved in the remaining three to four months. Finally, decision-making for large household purchases and the male partner’s health care was excluded from the analysis due to small sample size. The questions were only asked to men who were in a relationship (married or living together) and earned cash for employment in the 12 months preceding the baseline survey. Also, the survey did not measure emotional support provided to the FTM by the male partner during pregnancy (e.g., helping without being asked, telling her she is attractive, giving her massages (rubbing her back or massaging her feet), touching her belly, etc.). Using a comprehensive measure of involvement, further research needs to explore the effect of potential factors not included in this study (such as social norms, the provision of emotional support, and previous experience with own father) that can encourage or deter male involvement during pregnancy.

## Conclusions

Male partner participation during pregnancy is critical and affected by a myriad of factors. Knowledge that a woman needs four or more ANC visits, knowledge of one newborn danger sign, knowledge of one or more birth preparedness steps, relationship satisfaction, self-efficacy, and living in Lemba or Ndjili were positive correlates of involvement in ANC and birth preparedness, while always living in the health zone of residence and working throughout the year, seasonally, and occasionally were negative correlates. For shared decision-making, knowledge of two and three or more ANC benefits, gender-equitable attitudes, and the employment of both partners were positive correlates of involvement. Self-efficacy was a negative predictor of involvement in shared decisions. Addressing these determinants may improve male participation in maternal health. Using comprehensive approaches that improve men’s knowledge of maternal health, provide skills to strengthen their relationships with their partners, and improve couple communication is necessary to improve male involvement. Approaches focusing on encouraging male partner involvement should also include activities that build men’s self-efficacy and sensitization activities to reduce negative attitudes towards gender equality.

### Supplementary Information

Supplementary Material 1.

## Data Availability

The datasets used and/or analyzed during the current study are available from the corresponding author upon reasonable request.

## Abbreviations

ANC: Antenatal care
CFA: Confirmatory factor analysis
DRC: Democratic of the Congo
EFA: Exploratory factor analysis
FTM: First-time mother
GEM: Gender-equitable Men
IPV: Intimate partner violence
RAS: Relationship Assessment Scale
WHO: World Health Organization
