# Supplementary material for: Involving men in pregnancy: a cross-sectional analysis of the role of self-efficacy, gender-equitable attitudes, relationship dynamics and knowledge among men in Kinshasa
Source: BMC Pregnancy Childbirth. 2024 Jun 26;24:444. doi: 10.1186/s12884-024-06638-1 (PMC11202384; doi:10.1186/s12884-024-06638-1)
Supplement: Supplementary file 1 — Supplementary Material 1. [file 12884_2024_6638_MOESM1_ESM.pdf]

Supplemental Tables

Table S1: Fit indices obtained from the confirmatory factor analysis of the male involvement indexes, the gender equitable men's scale and the relationship assessment scale, Kinshasa 2018

Fit indices	Confirmatory Factor Analysis Model Fit			Cut-off points [29, 30]
	Male involvement index	Gender equitable men's scale	Relationship assessment scale	
RMSEA	0.057	0.048	0.061	< 0.05 – close fit<br>< 0.08 – acceptable fit
CFI	0.970	0.940	0.980	> 0.95 – good fit<br>> 0.90 – acceptable fit
TLI	0.958	0.918	0.968	> 0.95 – good fit<br>> 0.90 – acceptable fit
SRMR	0.047	0.037	0.026	< 0.05 – close fit<br>< 0.08 – acceptable fit
P close	0.114	0.626	0.126	< 0.05 – poor fit

Note: CFI - Comparative fit index; RMSEA - Root mean squared error of approximation; SRMR - Standardized root mean square residual; TLI - Tucker-Lewis Index

Table S2: Percent distribution of male partners of first-time mothers age 15 – 24 by their agreement with items comprising the Gender-equitable Men's (GEM) scale, Kinshasa 2018

Components of the GEM Scale	No.	Percent
**A woman's most important role is to take care of her home and cook for her family**		
Totally Agree	1,049	62.7
Partially	181	10.8
Disagree	444	26.5
**Men need sex more than women do**		
Totally Agree	772	46.1
Partially	310	18.5
Disagree	592	35.4
**You don't talk about sex, you just do it**		
Totally Agree	361	21.6
Partially	208	12.4
Disagree	1,105	66.0
**There are times when a woman deserves to be beaten**		
Totally Agree	437	26.1
Partially	315	18.8
Disagree	922	55.1
**Changing diapers, giving a bath, and feeding kids is the mother's responsibility**		
Totally Agree	1,041	62.2
Partially	238	14.2
Disagree	395	23.6
**It is a woman's responsibility to avoid getting pregnant**		
Totally Agree	592	35.4
Partially	329	19.7
Disagree	753	45.0
**A man should have the final word about decisions in his home**		

Components of the GEM Scale	No.	Percent
Totally Agree	1,277	76.3
Partially	224	13.4
Disagree	173	10.3
**Men are always ready to have sex**		
Totally Agree	775	46.3
Partially	333	19.9
Disagree	566	33.8
**A woman should tolerate violence to keep her family together**		
Totally Agree	615	36.7
Partially	315	18.8
Disagree	744	44.4
**I would be outraged if my wife asked me to use a condom** ‡		
Totally Agree	431	25.7
Partially	231	13.8
Disagree	1,012	60.5
**A man and a woman should decide together what type of contraceptive to use** † ‡		
Totally Agree	1,389	83.0
Partially	189	11.3
Disagree	96	5.7
**I would never have a gay friend** ‡		
Totally Agree	1,276	76.2
Partially	97	5.8
Disagree	301	18.0
**If someone insults me, I will defend my reputation, with force if I have to**		
Totally Agree	348	20.8
Partially	256	15.3
Disagree	1,070	63.9
**To be a man, you need to be tough**		
Totally Agree	745	44.5
Partially	292	17.4
Disagree	637	38.1
**Men should be embarrassed if they are unable to get an erection during sex** ‡		
Totally Agree	1,255	75.0
Partially	180	10.8
Disagree	239	14.3
**A couple should decide together if they want to have children** † ‡		
Totally Agree	1,460	87.2
Partially	142	8.5
Disagree	72	4.3
**A man should know what his partner likes during sex** † ‡		
Totally Agree	1,463	87.4
Partially	170	10.2
Disagree	41	2.4
N	1,674	100.0

Source: Momentum 2018 Baseline Survey

† - items were reversed coded such that a higher score supports gender equality

‡ - items had factor loadings less than 0.3, thus were dropped from the final scale

Column percentages may not add up to 100 due to rounding.

Table S3: Percent distribution of male partners of first-time mothers age 15 – 24 by the components of the generalized self-efficacy scale, Kinshasa 2018

Components of the Generalized Self-efficacy Scale	No.	Percent
**I can always manage to solve difficult problems if I try hard enough**		
Not at all	32	1.9
Hardly true	73	4.4
Moderately true	349	20.8
Always true	1,220	72.9
**If someone opposes me, I can find the means and ways to get what I want**		
Not at all	100	6.0
Hardly true	218	13.0
Moderately true	438	26.2
Always true	918	54.8
**It is easy for me to stick to my aims and accomplish my goals**		
Not at all	77	4.6
Hardly true	179	10.7
Moderately true	372	22.2
Always true	1,046	62.5
**I am confident that I could deal efficiently with unexpected events**		
Not at all	58	3.5
Hardly true	183	10.9
Moderately true	471	28.1
Always true	962	57.5
**Thanks to my resourcefulness, I know how to handle unforeseen situations**		
Not at all	246	14.7
Hardly true	389	23.2
Moderately true	419	25.0
Always true	620	37.0
**I can solve most problems if I invest the necessary effort.**		
Not at all	25	1.5
Hardly true	81	4.8
Moderately true	420	25.1
Always true	1,148	68.6
**I can remain calm when facing difficulties because I can rely on my coping ability**		
Not at all	53	3.2
Hardly true	139	8.3
Moderately true	413	24.7
Always true	1,069	63.9
**When I am confronted with a problem, I can usually find several solutions**		
Not at all	85	5.1
Hardly true	142	8.5
Moderately true	446	26.6
Always true	1,001	59.8
**If I am in trouble, I can usually think of a solution**		
Not at all	27	1.6
Hardly true	66	3.9
Moderately true	376	22.5
Always true	1,205	72.0
**I can usually handle whatever comes my way**		
Not at all	73	4.4
Hardly true	204	12.2

Components of the Generalized Self-efficacy Scale	No.	Percent
Moderately true	397	23.7
Always true	1,000	59.7
**Total**	**1,674**	**100.0**

Source: Momentum 2018 Baseline Survey
Column percentages may not add up to 100 due to rounding.

Table S4: Percent distribution of male partners of first-time mothers age 15 – 24 by the components of the relationship satisfaction scale, Kinshasa 2018

Components of the Relationship Assessment Scale	No.	Percent
**How well does (NAME OF FTM) meet your needs?**		
1 (low satisfaction)	54	3.2
2	53	3.2
3	340	20.3
4	418	25.0
5 (high satisfaction)	809	48.3
**In general, how satisfied are you with your relationship?**		
1 (low satisfaction)	49	2.9
2	49	2.9
3	246	14.7
4	408	24.4
5 (high satisfaction)	922	55.1
**How good is your relationship compared to most?**		
1 (low satisfaction)	45	2.7
2	54	3.2
3	253	15.1
4	375	22.4
5 (high satisfaction)	947	56.6
**How often do you wish you had not gotten into this relationship?** †		
1 (low satisfaction)	1,156	69.1
2	224	13.4
3	119	7.1
4	54	3.2
5 (high satisfaction)	121	7.2
**To what extent has your relationship met your original expectations?**		
1 (low satisfaction)	111	6.6
2	101	6.0
3	324	19.4
4	393	23.5
5 (high satisfaction)	745	44.5
**How much do you love (NAME OF FTM)?**		
1 (low satisfaction)	35	2.1
2	28	1.7
3	133	7.9
4	318	19.0
5 (high satisfaction)	1,160	69.3
**How many problems are there in your relationship?** †		
1 (low satisfaction)	933	55.7
2	369	22.0
3	209	12.5

Components of the Relationship Assessment Scale	No.	Percent
4	79	4.7
5 (high satisfaction)	84	5.0
Total	1,674	100.0

Source: Momentum 2018 Baseline Survey
† - items were reversed coded such that a higher score supports relationship satisfaction
Column percentages may not add up to 100 due to rounding.

Table S5: Description of socio-demographic variables used as control variables

Variable name	Categories	Values/Range
**Age of the male partner**	15-24 years	0 (reference group)
	25+ years	1
**Level of education of the male partner**	Lower than secondary	0 (reference group)
	Secondary complete	1
	Higher	2
**Marital status of the male partner**	Never married/Ever married	0 (reference group)/1
**Ethnicity of the male partner**	Bakongo	1 (reference group)
	Bas Kasai & Kwilu-Kwango	2
	Kasai/Katana/Tanganyika	3
	Other	4
**Health zone**	Bumbu	1 (reference group)
	Kingasani	2
	Lemba	3
	Masina 1	4
	Matete	5
	Ndjili	6
**Duration of residence in the health zone**	< 5 years	1 (reference group)
	5+ years	2
	Always	3
	Visitor	4
**Household wealth of the male partner**	Low	0 (reference group)
	Medium	1
	High	2
**Number of children of the male partner**	0	0 (reference group)
	1	1
	2+	2
**Employment (in past 12 months) of the male partner**	Unemployed	0 (reference group)
	Employed with cash earnings	1
	Employed without cash earnings	2
**Duration of employment (in past 12 months) of the male partner**	Unemployed	0 (reference group)
	Throughout	1
	Seasonally	2
	Occasionally	3
**Both partners worked for cash**	No/Yes	0 (reference group)/1
**Age difference between FTM and male partner**	MP younger/<5 years older	0 (reference group)
	5 - 9 years older	1
	10+ years older	2

Table S6: Percent distribution of male partners of first-time mothers age 15 – 24 by presence of missing data, Kinshasa 2018

	Male partners without missing data			
	**No**	**Yes**	**Total**	
**Age distribution**				
15-24	30.5	33.1	32.7	
25+	69.5	66.9	67.3	
**Level of education**				
None/Primary/Sec incomplete	35.1	32.7	33.1	
Secondary complete/higher	47.9	45.8	46.1	
Higher	17.0	21.5	20.7	
**Marital Status**				***
Never married	74.4	87.3	85.1	
Ever married	25.6	12.7	14.9	
**Ethnicity**				
Bakongo	27.2	30.5	29.9	
Bas Kasai & Kwilu-Kwango	43.3	41.7	42.0	
Kasai/Katana/Tanganyika	16.7	12.3	13.1	
Other	12.8	15.5	15.1	
**Health zone of residence**				***
Bumbu	8.2	12.9	12.1	
Kingasani	30.5	22.3	23.7	
Lemba	8.5	14.7	13.6	
Masina1	30.5	19.8	21.7	
Matete†	7.5	11.4	10.7	
Ndjili	14.8	18.8	18.1	
**Duration of residence in the health zone**				*
<5 years	47.5	42.4	43.3	
5+ years	19.7	17.7	18.0	
Always	27.9	37.0	35.4	
Visitor†	4.9	2.9	3.2	
**No of children ever fathered**				
0	73.4	73.6	73.6	
1	16.1	17.4	17.2	
2+	10.5	9.0	9.3	
**Household wealth**				
Low	31.5	34.5	34.0	
Middle	33.8	32.8	33.0	
High	34.8	32.7	33.1	
**Employment in the past 12 months**				
No Work	11.8	10.7	10.9	
Work for cash only	82.0	79.6	80.0	
Work but not paid, worked for kind or cash and kind†	6.2	9.7	9.1	
**Duration of employment**				
Unemployed	15.4	15.2	15.2	
Throughout the year	51.8	51.8	51.8	
Seasonally	13.8	14.7	14.6	
Occasionally	19.0	18.3	18.4	
.				
Total	100.0	100.0	100.0	
N	305	1,674	1,766	

Note: SD - Standard deviation
† The number of partners with missing data in the indicated categories was less than 25
***<0.001; ** <0.01; *<0.05

Table S7: Levels of involvement in shared decisions and antenatal care and birth preparedness activities by male partners' knowledge, co-parental relationship, self-efficacy, and gender-equitable attitudes, Kinshasa 2018

	Male involvement in ANC and birth preparedness					Male involvement in shared decisions				
	Level of involvement					Level of involvement				
**Independent Variables**	**Low**	**Medium**	**High**	**Total**		**Low**	**Medium**	**High**	**Total**	
**Knowledge of ANC benefits**					**					**
0 – 1	51.3	22.5	26.2	100.0		64.8	17.4	17.8	100.0	
2	37.0	28.2	34.8	100.0		56.9	16.6	26.5	100.0	
3+	43.4	22.5	34.1	100.0		52.7	18.4	29.0	100.0	
**Knowledge of the number of ANC visits**					***					
<4 times	50.6	25.6	23.8	100.0		58.4	18.5	23.1	100.0	
≥ 4 times	38.4	23.7	37.9	100.0		55.0	17.1	27.8	100.0	
**Knowledge of the start of ANC**										
After first trimester	41.8	25.9	32.3	100.0		57.2	17.8	25.0	100.0	
During first trimester	43.7	22.7	33.5	100.0		55.2	17.4	27.5	100.0	
**Knowledge about danger signs for mother**					***					***
0 †	70.9	13.6	15.5	100.0		68.0	13.6	18.4	100.0	
1	38.0	26.9	35.1	100.0		61.7	19.8	18.5	100.0	
2	44.8	23.2	32.0	100.0		56.1	17.2	26.7	100.0	
3+	38.4	25.9	35.7	100.0		50.4	17.4	32.1	100.0	
**Knowledge about danger signs for newborns**					***					**
0 †	64.2	14.6	21.1	100.0		61.8	21.1	17.1	100.0	
1	43.1	23.5	33.4	100.0		62.3	15.5	22.1	100.0	
2	37.5	26.7	35.8	100.0		53.9	18.3	27.8	100.0	
3+	42.6	25.2	32.2	100.0		51.0	18.2	30.8	100.0	
**Knowledge of birth preparedness steps**					***					***
0 †	71.7	21.7	6.5	100.0		67.4	16.3	16.3	100.0	
1	48.6	26.1	25.3	100.0		59.1	17.3	23.6	100.0	
2	30.2	22.4	47.4	100.0		52.8	17.0	30.2	100.0	
3+	27.8	21.7	50.4	100.0		39.1	24.3	36.5	100.0	
**Past-year perpetuation of emotional violence ‡**										**
No	44.2	23.6	32.2	100.0		53.5	17.9	28.6	100.0	
Yes	41.7	21.1	37.2	100.0		64.9	15.3	19.8	100.0	
**Past-year perpetuation of physical violence ‡**					*					

	Male involvement in ANC and birth preparedness					Male involvement in shared decisions				
	Level of involvement					Level of involvement				
**Independent Variables**	**Low**	**Medium**	**High**	**Total**		**Low**	**Medium**	**High**	**Total**	
No	41.4	23.9	34.7	100.0		53.7	17.1	29.2	100.0	
Yes	48.7	21.8	29.6	100.0		58.7	18.1	23.2	100.0	
**Past-year perpetuation of sexual violence** ‡										*
No	43.4	23.3	33.3	100.0		54.7	17.2	28.1	100.0	
Yes	48.0	22.4	29.6	100.0		62.4	20.0	17.6	100.0	
.										
Total	42.7	24.4	32.9	100.0		55.7	17.5	26.8	100.0	
.										
Mean (SD)										
**Relationship satisfaction (range: 7 - 35)**	29.62 (5.49)	28.86 (4.87)	30.19 (4.87)	29.62 (5.06)	***	29.34 (5.23)	30.33 (4.38)	29.73 (5.09)	29.62 (5.09)	*
**Gender-equitable attitude (range: 11 - 33)**	21.86 (4.97)	21.70 (4.60)	21.26 (4.79)	21.64 (4.83)		21.08 (4.76)	21.83 (4.45)	22.70 (5.03)	21.64 (4.83)	***
**Perceived self-efficacy (range: 13 - 40)**	33.64 (5.22)	33.22 (4.20)	35.53 (3.67)	34.16 (4.61)	***	34.59 (4.54)	33.25 (4.71)	33.85 (4.60)	34.16 (4.61)	***
N	715	408	551	1,674		941	295	438	1,674	

Note: ANC – antenatal care; IPV: intimate partner violence; max – maximum value; SD – Standard deviation

Levels of involvement: Low = 0 shared decisions and 0 ANC and birth preparedness activities; Medium = 1 – 2 shared decisions and 1 – 3 ANC and birth preparedness activities; and High = 3 shared decisions and 4 – 7 ANC and birth preparedness activities.

† The number of partners with low, medium, and high involvement in at least one cell in the category was less than 25;

‡ Only men who had privacy during the interview were asked IPV questions (N=1,461) - ANC & Birth preparedness (Low (N=640); Medium (N=339); High (N=482)); Decisions (Low (N=809); Medium (N=225); High (N=397));

***<0.001; ** <0.01; *<0.05

Table S8: Results of multivariate linear regression models of male involvement in antenatal care and birth preparedness, Kinshasa 2018

	Male involvement in ANC and birth preparedness					
	Total sample			Total sample with interaction		
	**Beta**	**SE**	**95% CI**	**Beta**	**SE**	**95% CI**
**Knowledge of ANC benefits**						

	Male involvement in ANC and birth preparedness							
	Total sample				Total sample with interaction			
	Beta		SE	95% CI	Beta		SE	95% CI
0 - 1	[REF]				[REF]			
2	0.192		0.170	[-0.142, 0.526]	0.187		0.170	[-0.147, 0.521]
3+	0.040		0.176	[-0.306, 0.385]	0.052		0.176	[-0.294, 0.399]
**Knowledge of the number of ANC visits**								
<4 times	[REF]				[REF]			
≥ 4 times	0.5222	***	0.125	[0.277, 0.767]	0.523	***	0.125	[0.278, 0.768]
**Knowledge of the start of ANC**								
After first trimester	[REF]				[REF]			
During first trimester	-0.077		0.113	[-0.298, 0.144]	-0.081		0.113	[-0.302, 0.140]
**Knowledge about danger signs for mother**								
0	[REF]				[REF]			
1	0.490		0.262	[-0.023, 1.003]	0.467		0.261	[-0.046, 0.980]
2	0.101		0.258	[-0.405, 0.606]	0.092		0.258	[-0.413, 0.597]
3+	0.220		0.276	[-0.319, 0.760]	0.226		0.275	[-0.314, 0.765]
**Knowledge about danger signs for newborns**								
0	[REF]				[REF]			
1	0.462	*	0.233	[0.004, 0.919]	0.469		0.235	[0.009, 0.929]
2	0.415		0.243	[-0.063, 0.892]	0.436		0.244	[-0.042, 0.914]
3+	0.159		0.264	[-0.359, 0.678]	0.191		0.265	[-0.328, 0.710]
**Knowledge of birth preparedness steps**								
0	[REF]				[REF]			
1	0.737	**	0.266	[0.215, 1.260]	0.732	**	0.266	[0.210, 1.254]
2	1.964	***	0.283	[1.408, 2.520]	1.957	***	0.283	[1.402, 2.513]
3+	2.495	***	0.348	[1.812, 3.179]	2.464	***	0.349	[1.779, 3.149]
**Past-year perpetuation of emotional violence**								
No	[REF]				[REF]			
Yes	0.322		0.179	[-0.029, 0.673]	-0.643		0.931	[-2.469, 1.183]
**Past-year perpetuation of physical violence**								
No	[REF]				[REF]			
Yes	-0.195		0.140	[-0.471, 0.080]	-0.391		0.856	[-2.071, 1.288]
**Past-year perpetuation of sexual violence**								
No	[REF]				[REF]			
Yes	-0.133		0.211	[-0.546, 0.281]	-0.295		1.084	[-2.421, 1.831]
**Relationship satisfaction (max = 35)**	0.036	**	0.012	[0.012, 0.059]	-0.110	*	0.054	[-0.215, -0.004]
**Gender-equitable attitude (max = 33)**	-0.023		0.013	[-0.049, 0.002]	-0.222	**	0.075	[-0.368, -0.075]
**Perceived self-efficacy (max = 40)**	0.092	***	0.013	[0.066, 0.118]	0.093	***	0.013	[0.066, 0.119]

	Male involvement in ANC and birth preparedness							
	Total sample				Total sample with interaction			
	Beta		SE	95% CI	Beta		SE	95% CI
Interaction terms								
**Relationship sat. x gender-equitable attitude**					0.007	**	0.002	[0.002, 0.011]
**Relationship sat. x emotional IPV perpetration**					0.034		0.032	[-0.028, 0.097]
**Relationship sat. x physical IPV perpetration**					0.006		0.028	[-0.049, 0.061]
**Relationship sat. x sexual IPV perpetration**					0.006		0.038	[-0.069, 0.080]
Control Variables								
**Age distribution**								
15-24	[REF]				[REF]			
25+	-0.045		0.152	[-0.344, 0.253]	-0.057		0.152	[-0.356, 0.242]
**Level of education**								
Lower than secondary	[REF]				[REF]			
Secondary complete	-0.054		0.132	[-0.313, 0.205]	-0.064		0.132	[-0.324, 0.195]
Higher	0.183		0.174	[-0.158, 0.524]	0.174		0.174	[-0.167, 0.514]
**Marital Status**								
Never married	[REF]				[REF]			
Ever married	-0.260		0.170	[-0.595, 0.074]	-0.233		0.171	[-0.568, 0.102]
**Ethnicity**								
Bakongo	[REF]				[REF]			
Bas Kasai & Kwilu-Kwango	0.060		0.154	[-0.242, 0.361]	0.062		0.153	[-0.239, 0.362]
Kasai/Katana/Tanganyika	-0.193		0.201	[-0.587, 0.200]	-0.187		0.201	[-0.581, 0.206]
Other	0.019		0.184	[-0.343, 0.380]	0.021		0.184	[-0.341, 0.382]
**Health zone of residence**								
Bumbu	[REF]				[REF]			
Kingasani	0.268		0.235	[-0.194, 0.730]	0.318		0.236	[-0.144, 0.781]
Lemba	0.870	***	0.239	[0.400, 1.339]	0.931	***	0.240	[0.460, 1.401]
Masina1	0.103		0.230	[-0.347, 0.553]	0.154		0.230	[-0.298, 0.606]
Matete	-0.133		0.239	[-0.602, 0.337]	-0.072		0.240	[-0.543, 0.399]
Ndjili	1.482	***	0.223	[1.046, 1.919]	1.553	***	0.224	[1.113, 1.993]
**Duration of residence in the health zone**								
<5 years	[REF]				[REF]			
5+ years	0.050		0.159	[-0.261, 0.362]	0.054		0.159	[-0.257, 0.366]
Always	-0.292	*	0.190	[-0.545, -0.039]	-0.294*	*	0.130	[-0.548, -0.040]
Visitor	0.182		0.338	[-0.482, 0.846]	0.142		0.339	[-0.523, 0.806]
**No of children ever fathered**								
0	[REF]				[REF]			
1	-0.227		0.152	[-0.525, 0.070]	-0.221		0.151	[-0.518, 0.076]

	Male involvement in ANC and birth preparedness							
	Total sample				Total sample with interaction			
	Beta		SE	95% CI	Beta		SE	95% CI
2+	-0.203		0.210	[-0.614, 0.209]	-0.211		0.210	[-0.623, 0.200]
**Household wealth**								
Low	[REF]				[REF]			
Middle	-0.105		0.139	[-0.377, 0.168]	-0.094		0.139	[-0.366, 0.179]
High	0.249		0.150	[-0.044, 0.543]	0.259		0.150	[-0.035, 0.552]
**Employment in the past 12 months**								
No Work	[REF]				[REF]			
Work for cash only	0.852	*	0.407	[0.054, 1.650]	0.760		0.408	[-0.041, 1.561]
Work but not paid, worked for kind or cash and kind	0.138		0.313	[-0.477, 0.753]	0.107		0.313	[-0.508, 0.722]
**Duration of employment**								
Unemployed	[REF]				[REF]			
Throughout the year	-0.901	*	0.369	[-1.624, -0.177]	-0.839	*	0.369	[-1.563, -0.114]
Seasonally	-1.325	***	0.370	[-2.051, -0.600]	-1.216	**	0.372	[-1.946, -0.487]
Occasionally	-1.078	**	0.382	[-1.828, -0.328]	-1.008	**	0.383	[-1.759, -0.257]
**Dual employment**								
No	[REF]				[REF]			
Yes	-0.098		0.130	[-0.354, 0.158]	-0.093		0.130	[-0.349, 0.162]
**Relative age difference between the FTM and MP**								
MP younger/<5 years older	[REF]				[REF]			
5 - 9 years older	0.021		0.148	[-0.269, 0.311]	0.048		0.148	[-0.349, 0.162]
10+ years older	0.059		0.190	[-0.314, 0.432]	0.069		0.190	[-0.305, 0.442]
.								
Constant	-3.543	***	0.785	[-5.083, -2.004]	0.078		1.762	[-2.675, 4.238]
Observations			1,461				1,461	
adjusted R-squared			0.240				0.245	
VIF			1.26					

Note: ANC – antenatal care; Beta – Unstandardized adjusted coefficient; SE- Standard Error; CI – confidence interval; IPV – intimate partner violence; max – maximum; ref – reference

***<0.001; ** <0.01; *<0.05

Table S9: Results of multivariate linear regression models of male involvement in shared decisions about pregnancy, Kinshasa 2018

	Male involvement in shared decisions							
	Total sample				Total sample with interaction			
	Beta		SE	95% CI	Beta		SE	95% CI
**Knowledge of ANC benefits**								
0 - 1	[REF]				[REF]			
2	0.195	*	0.085	[0.028, 0.363]	0.200	*	0.085	[0.032, 0.367]
3+	0.218	*	0.088	[0.045, 0.391]	0.231	**	0.088	[0.058, 0.405]
**Knowledge of the number of ANC visits**								
<4 times	[REF]				[REF]			
≥ 4 times	0.097		0.063	[-0.026, 0.219]	0.094		0.063	[-0.029, 0.217]
**Knowledge of the start of ANC**								
After first trimester	[REF]				[REF]			
During first trimester	-0.013		0.056	[-0.124, 0.098]	-0.014		0.056	[-0.125, 0.097]
**Knowledge about danger signs for mother**								
0	[REF]				[REF]			
1	-0.017		0.131	[-0.274, 0.240]	-0.016		0.131	[-0.273, 0.241]
2	0.127		0.129	[-0.126, 0.381]	0.132		0.129	[-0.121, 0.385]
3+	0.227		0.138	[-0.043, 0.497]	0.238		0.138	[-0.033, 0.508]
**Knowledge about danger signs for newborns**								
0	[REF]				[REF]			
1	0.089		0.117	[-0.140, 0.318]	0.072		0.118	[-0.159, 0.303]
2	0.099		0.122	[-0.140, 0.338]	0.089		0.122	[-0.151, 0.329]
3+	0.010		0.132	[-0.250, 0.269]	0.006		0.133	[-0.255, 0.266]
**Knowledge of birth preparedness steps**								
0	[REF]				[REF]			
1	0.040		0.133	[-0.221, 0.302]	0.032		0.133	[-0.229, 0.294]
2	0.165		0.142	[-0.113, 0.444]	0.159		0.142	[-0.119, 0.438]
3+	0.275		0.175	[-0.067, 0.618]	0.253		0.175	[-0.091, 0.596]
**Past-year perpetuation of emotional violence**								
No	[REF]				[REF]			
Yes	-0.148		0.090	[-0.324, 0.028]	0.104		0.466	[-0.811, 1.019]
**Past-year perpetuation of physical violence**								
No	[REF]				[REF]			
Yes	-0.044		0.070	[-0.182, 0.094]	-0.175		0.429	[-1.017, 0.667]
**Past-year perpetuation of sexual violence**								
No	[REF]				[REF]			
Yes	0.025		0.106	[-0.182, 0.232]	-1.023		0.543	[-2.089, 0.042]
**Relationship satisfaction (max = 35)**	0.001		0.006	[-0.011, 0.012]	-0.054	*	0.027	[-0.107, -0.001]
**Gender-equitable attitude (max = 33)**	0.034	***	0.007	[0.021, 0.047]	-0.041		0.037	[-0.115, 0.032]

	Male involvement in shared decisions							
	Total sample				Total sample with interaction			
	Beta		SE	95% CI	Beta		SE	95% CI
**Perceived self-efficacy (max = 40)**	-0.016	*	0.007	[-0.029, -0.002]	-0.016	*	0.007	[-0.029, -0.003]
Interaction terms								
**Relationship sat. x gender-equitable attitude**					0.002	*	0.001	[0.000, 0.005]
**Relationship sat. x emotional IPV perpetration**					-0.009		0.016	[-0.040, 0.023]
**Relationship sat. x physical IPV perpetration**					0.004		0.014	[-0.023, 0.032]
**Relationship sat. x sexual IPV perpetration**					0.037	*	0.019	[0.000, 0.075]
Control Variables								
**Age distribution**								
15-24	[REF]				[REF]			
25+	-0.044		0.076	[-0.193, 0.106]	-0.055		0.076	[-0.204, 0.095]
**Level of education**								
Lower than secondary	[REF]				[REF]			
Secondary complete	-0.070		0.066	[-0.200, 0.060]	-0.069		0.066	[-0.198, 0.061]
Higher	0.094		0.087	[-0.077, 0.264]	0.095		0.087	[-0.075, 0.266]
**Marital Status**								
Never married	[REF]				[REF]			
Ever married	0.059		0.085	[-0.108, 0.227]	0.069		0.085	[-0.099, 0.237]
**Ethnicity**								
Bakongo	[REF]				[REF]			
Bas Kasai & Kwilu-Kwango	-0.054		0.077	[-0.205, 0.097]	-0.054		0.077	[-0.204, 0.097]
Kasai/Katana/Tanganyika	-0.050		0.100	[-0.247, 0.148]	-0.054		0.101	[-0.251, 0.144]
Other	-0.048		0.092	[-0.229, 0.133]	-0.047		0.092	[-0.229, 0.134]
**Health zone of residence**								
Bumbu	[REF]				[REF]			
Kingasani	0.160		0.118	[-0.071, 0.391]	0.181		0.118	[-0.051, 0.413]
Lemba	-0.232		0.120	[-0.468, 0.003]	-0.241		0.120	[-0.450, 0.021]
Masina1	-0.019		0.115	[-0.244, 0.207]	-0.012		0.115	[-0.238, 0.215]
Matete	0.003		0.120	[-0.232, 0.239]	0.019		0.120	[-0.217, 0.255]
Ndjili	-0.135		0.112	[-0.353, 0.084]	-0.108		0.112	[-0.329, 0.112]
**Duration of residence in the health zone**								
<5 years	[REF]				[REF]			
5+ years	-0.132		0.080	[-0.288, 0.024]	-0.132		0.080	[-0.288, 0.024]
Always	0.006		0.066	[-0.121, 0.133]	-0.003		0.065	[-0.130, 0.125]
Visitor	-0.002		0.169	[-0.335, 0.331]	-0.001		0.170	[-0.334, 0.332]
**No of children ever fathered**								
0	[REF]				[REF]			

	Male involvement in shared decisions							
	Total sample				Total sample with interaction			
	Beta		SE	95% CI	Beta		SE	95% CI
1	-0.125		0.076	[-0.274, 0.024]	-0.126		0.076	[-0.275, 0.023]
2+	0.044		0.105	[-0.251, 0.162]	-0.051		0.105	[-0.257, 0.155]
**Household wealth**								
Low	[REF]				[REF]			
Middle	0.080		0.070	[-0.057, 0.216]	0.086		0.070	[-0.051, 0.222]
High	0.068		0.075	[-0.079, 0.215]	0.072		0.075	[-0.075, 0.219]
**Employment in the past 12 months**								
No Work	[REF]				[REF]			
Work for cash only	-0.059		0.204	[-0.458, 0.341]	-0.082		0.205	[-0.484, 0.319]
Work but not paid, worked for kind or cash and kind	0.054		0.157	[-0.254, 0.362]	0.050		0.157	[-0.258, 0.358]
**Duration of employment**								
Unemployed	[REF]				[REF]			
Throughout the year	0.032		0.185	[-0.330, 0.395]	0.057		0.185	[-0.306, 0.420]
Seasonally	0.112		0.185	[-0.251, 0.476]	0.147		0.186	[-0.218, 0.513]
Occasionally	0.157		0.191	[-0.219, 0.532]	0.182		0.192	[-0.194, 0.558]
**Dual employment**								
No	[REF]				[REF]			
Yes	0.140	*	0.065	[0.012, 0.268]	0.143	*	0.065	[0.015, 0.272]
**Relative age difference between the FTM and MP**								
MP younger/<5 years older	[REF]				[REF]			
5 - 9 years older	0.097		0.074	[-0.048, 0.242]	0.112		0.074	[-0.034, 0.257]
10+ years older	0.159		0.095	[-0.028, 0.346]	0.173		0.095	[-0.014, 0.360]
.								
Constant	0.113		0.393	[-0.658, 0.885]	1.777	*	0.883	[0.045, 3.509]
N			1,461				1,461	
adjusted R-squared			0.100				0.11	
VIF			1.26					

Note: ANC – antenatal care; Beta – Unstandardized adjusted coefficient; SE- Standard Error; CI – confidence interval; IPV – intimate partner violence; max – maximum; ref – reference

***<0.001; ** <0.01; *<0.05
